# Supplementary figures and images for: Convergent adaptation of Saccharomyces uvarum to sulfite, an antimicrobial preservative widely used in human-driven fermentations
Source: PLoS Genet. 2021 Nov 11;17(11):e1009872. doi: 10.1371/journal.pgen.1009872 (PMC8631656; doi:10.1371/journal.pgen.1009872)

A

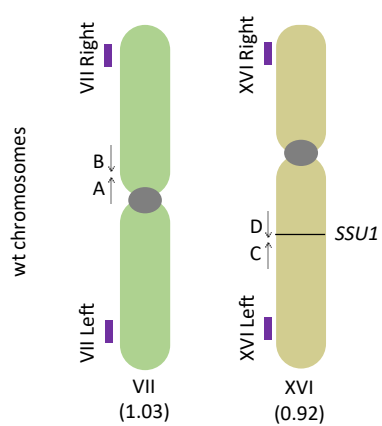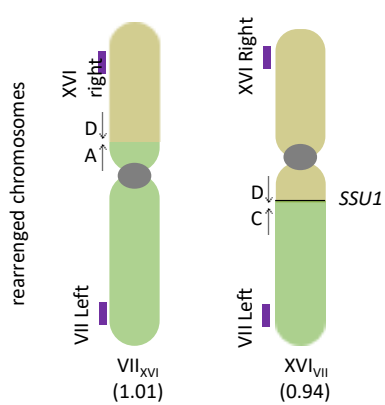

B

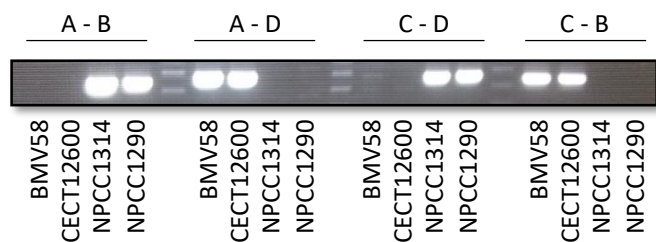

C

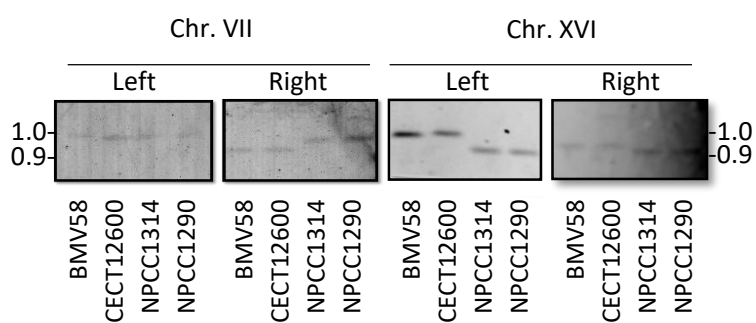

Supplement: S1 Fig — (A) A schematic representation of the chromosomal location of primers (arrows) and probes (purple rectangles) used to detect wild type (VII and XVI) and recombinant (VIIXVI and XVIVII) chromosomes. Chromosomal size in Mbp is indicated in brackets. (B) PCR amplification used to test for the presence of wild type chromosomes VII (primers A-B) and XVI (primers C-D) or recombinant chromosomes VIIXVI (primers D-B) and XVIVII (primers C-A). (C) Southern blots with chromosome VII and XVI left and right probes performed in genomic DNA obtained from BMV58, CECT12600, NPCC1290, and NPCC1314 S. uvarum strains. DNA fragment size is indicated in Mbp. (PDF) [file pgen.1009872.s006.pdf]

# Pairwise genetic distances (BMV58)

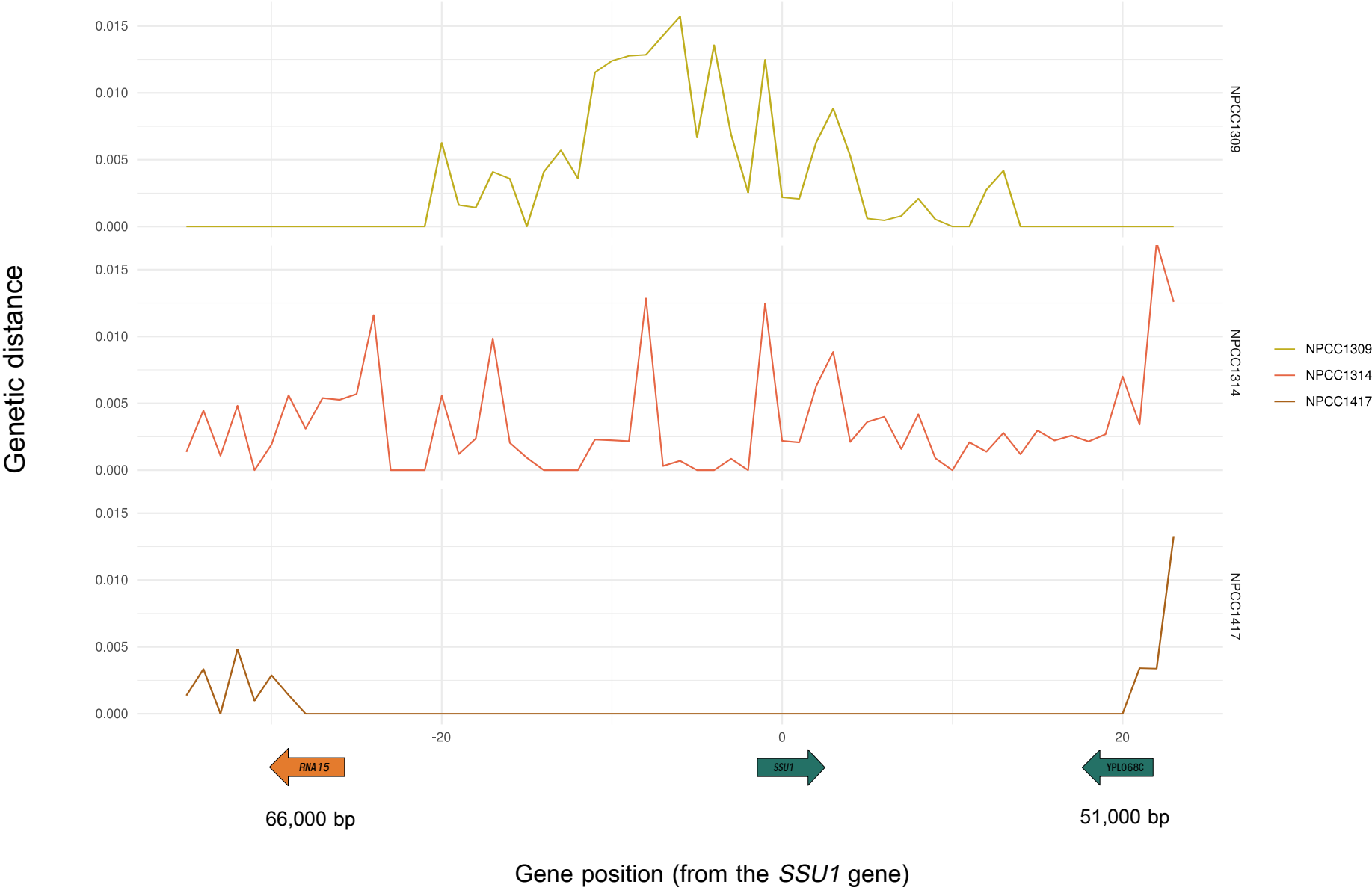

Supplement: S2 Fig — Pairwise genetic distances of the genes surrounding the SSU1 promoter were calculated and represented in this study using BMV58 as reference. The x-axis represents the gene position using SSU1 as reference (position 0). Green genes correspond to genes from the reference chromosome XVI and the orange gene corresponds to the reference chromosome VII. (PDF) [file pgen.1009872.s007.pdf]

## Pairwise genetic distances (BMV58)

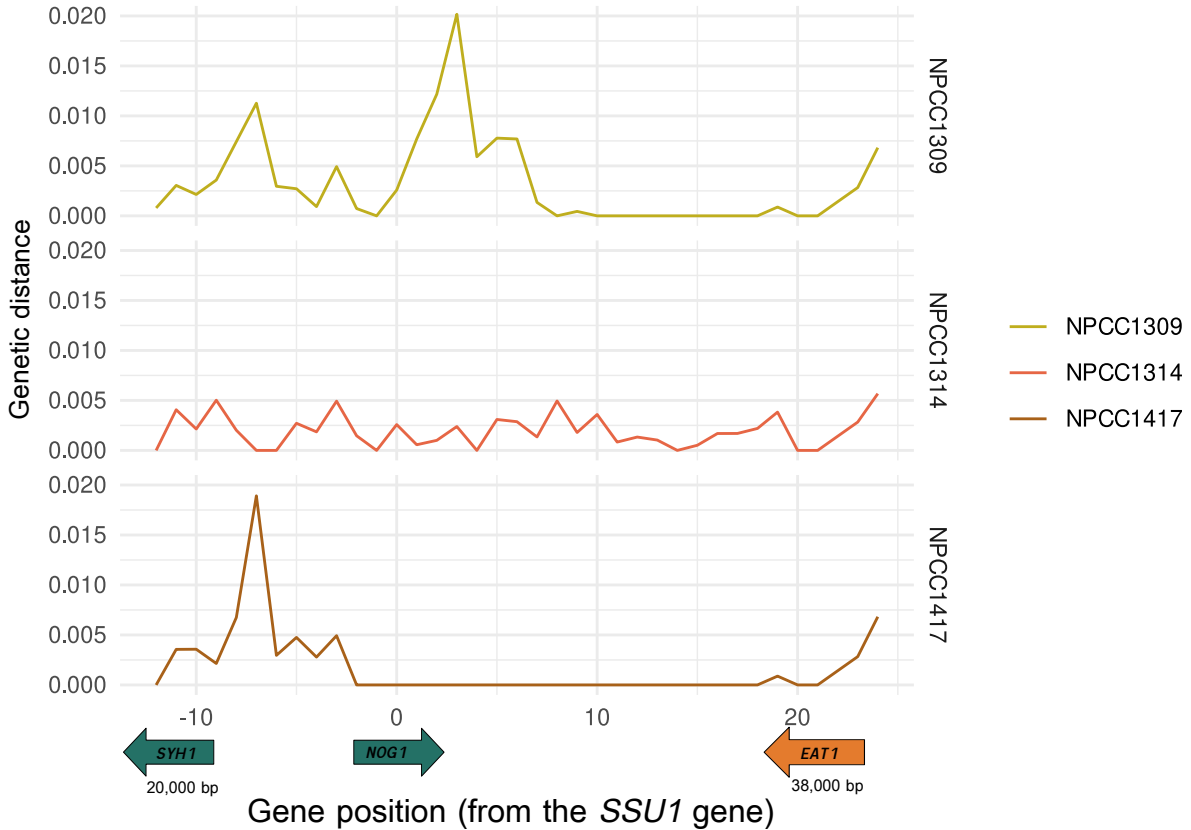

Supplement: S3 Fig — Pairwise genetic distances of the genes surrounding the SSU1 promoter were calculated and represented in this study using BMV58 as reference. The x-axis represents the gene position using SSU1 as reference (position 0). Green genes correspond to genes from the reference chromosome XVI and the orange gene corresponds to the reference chromosome VII. (PDF) [file pgen.1009872.s008.pdf]

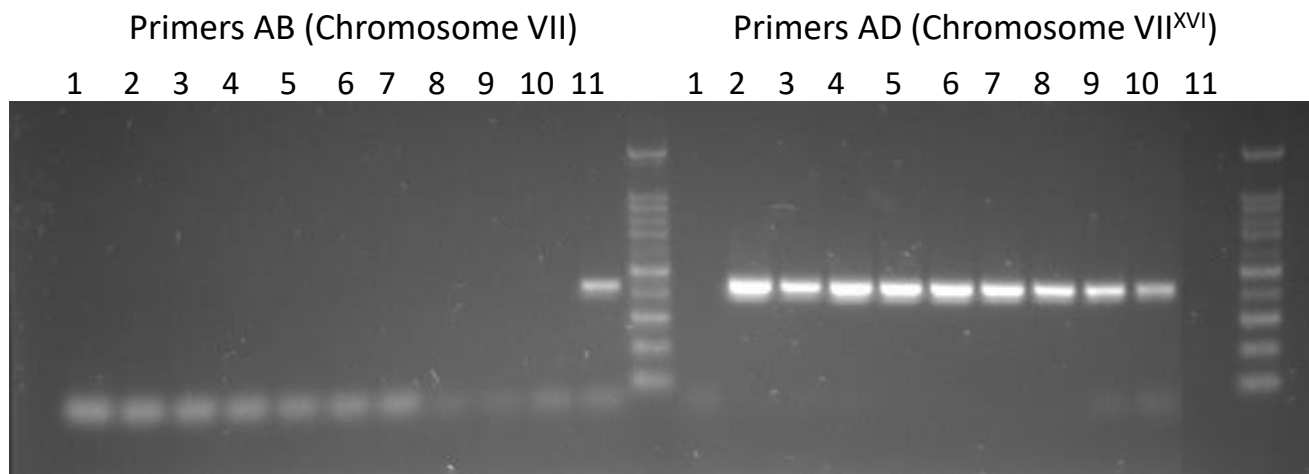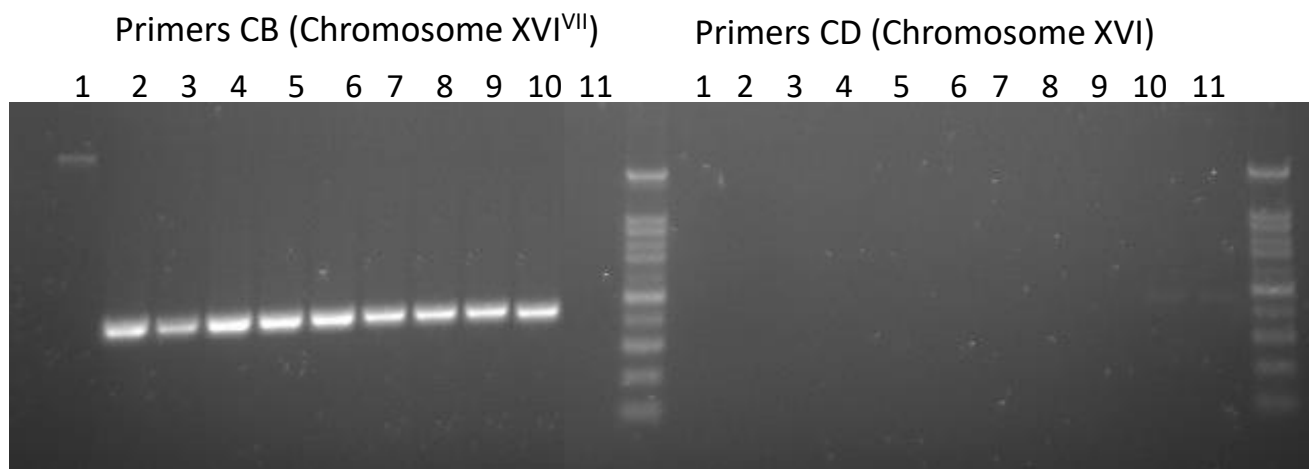

# Number Strain

- 1 T73 (S.c.)
- 2 CETC1884
- 3 CECT12600
- 4 CECT12627
- 5 CECT12922
- 6 CECT12930
- 7 BMV58
- 8 NPCC1417
- 9 NPCC1418
- 10 NPCC1419
- 11 BR6-2 (XI tXVI)

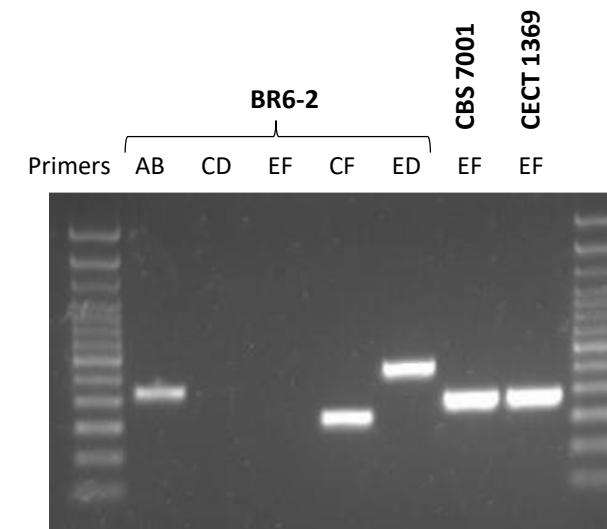

Supplement: S8 Fig — (PDF) [file pgen.1009872.s013.pdf]

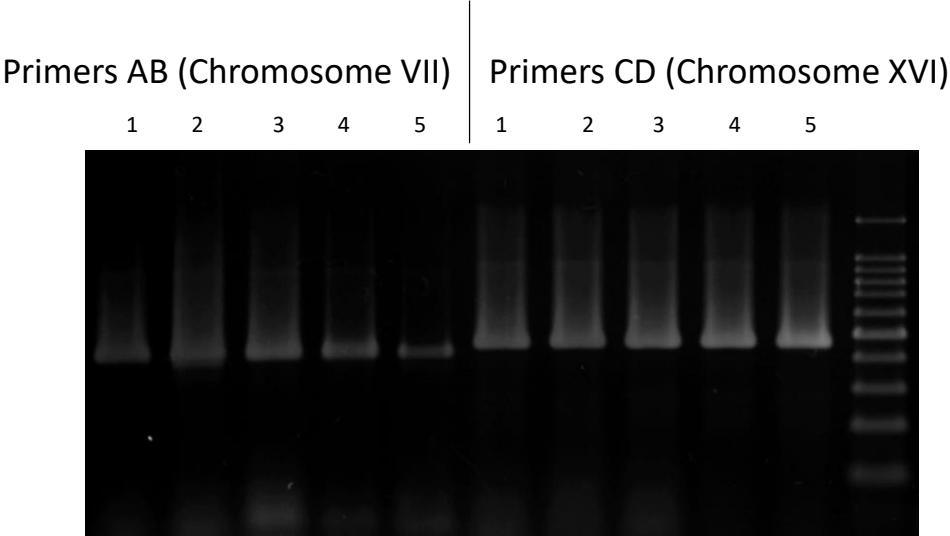

| Number | Strain   |
|--------|----------|
| 1      | NPCC1319 |
| 2      | NPCC1411 |
| 3      | NPCC1412 |
| 4      | NPCC1413 |
| 5      | NPCC1414 |
| 6      | NPCC1416 |
| 7      | NPCC1427 |
| 8      | NPCC1428 |

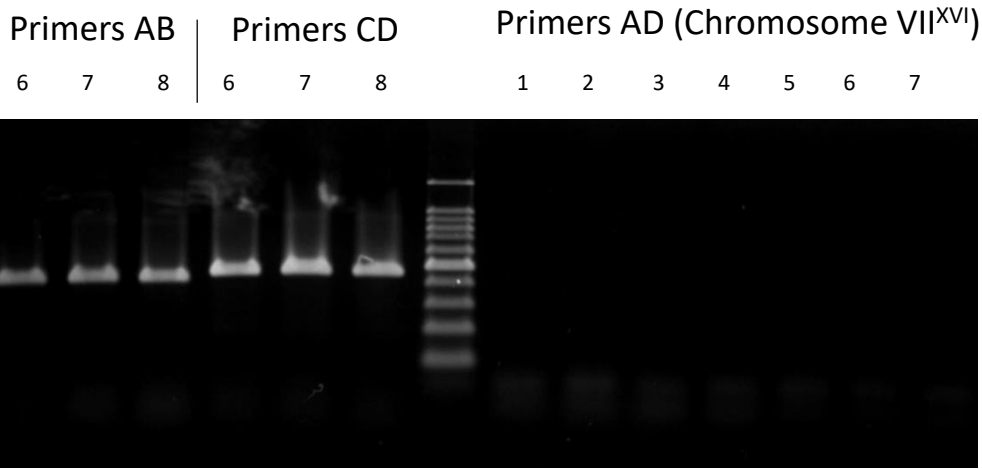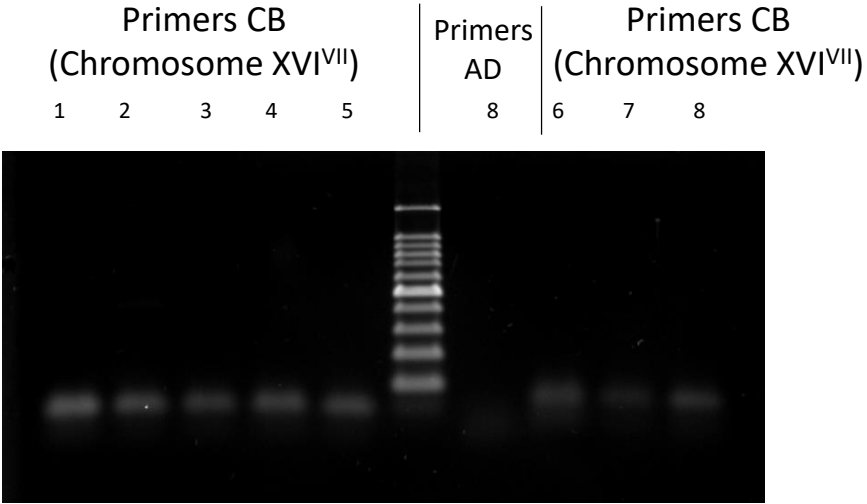

Supplement: S9 Fig — (PDF) [file pgen.1009872.s014.pdf]

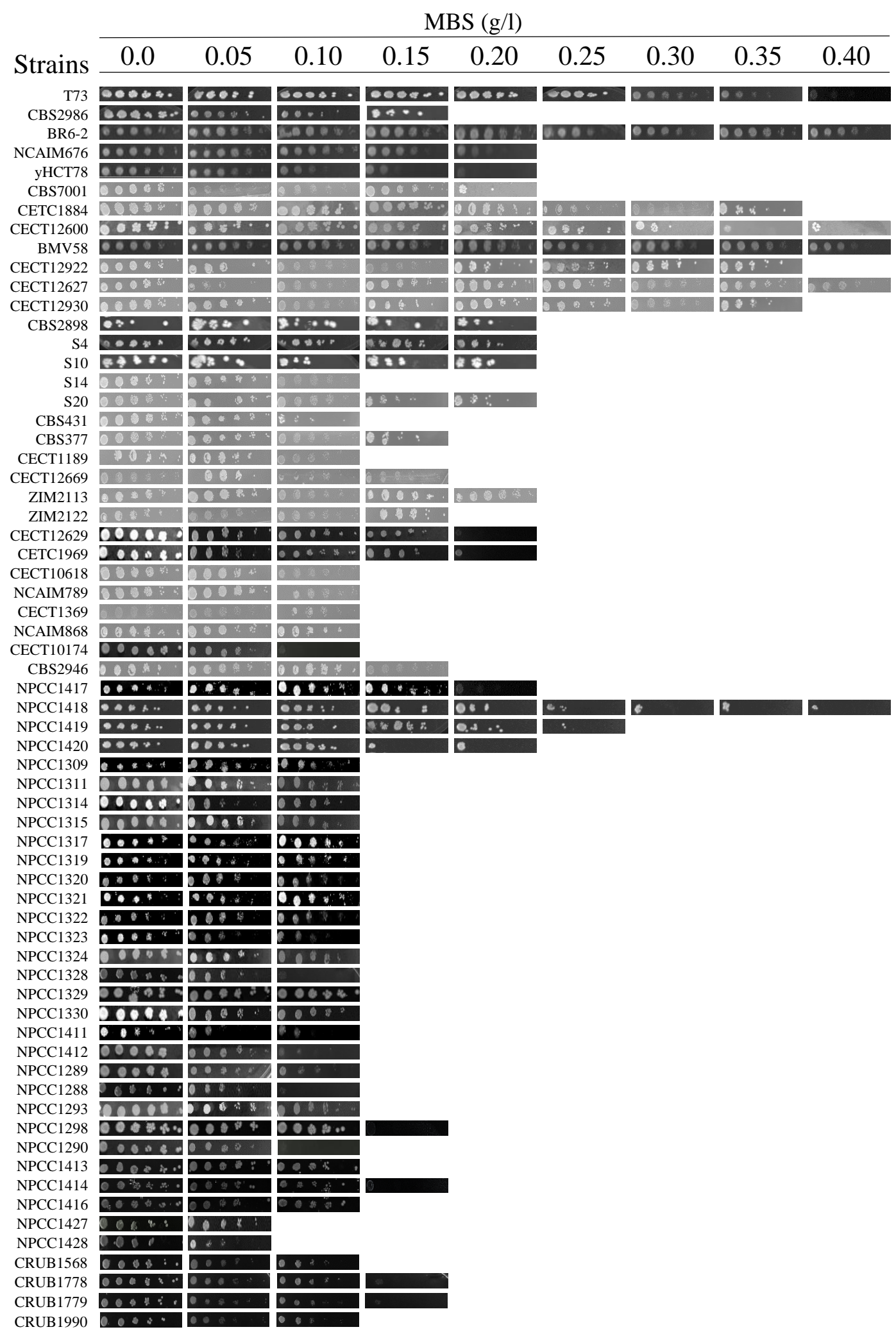

Supplement: S11 Fig — Nine MBS concentrations (0.0–0.4 g/l) were evaluated. (PDF) [file pgen.1009872.s016.pdf]
