# Supplementary material for: Convergent adaptation of Saccharomyces uvarum to sulfite, an antimicrobial preservative widely used in human-driven fermentations
Source: PLoS Genet. 2021 Nov 11;17(11):e1009872. doi: 10.1371/journal.pgen.1009872 (PMC8631656; doi:10.1371/journal.pgen.1009872)
Supplement: S4 Fig — (PDF) [file pgen.1009872.s009.pdf]

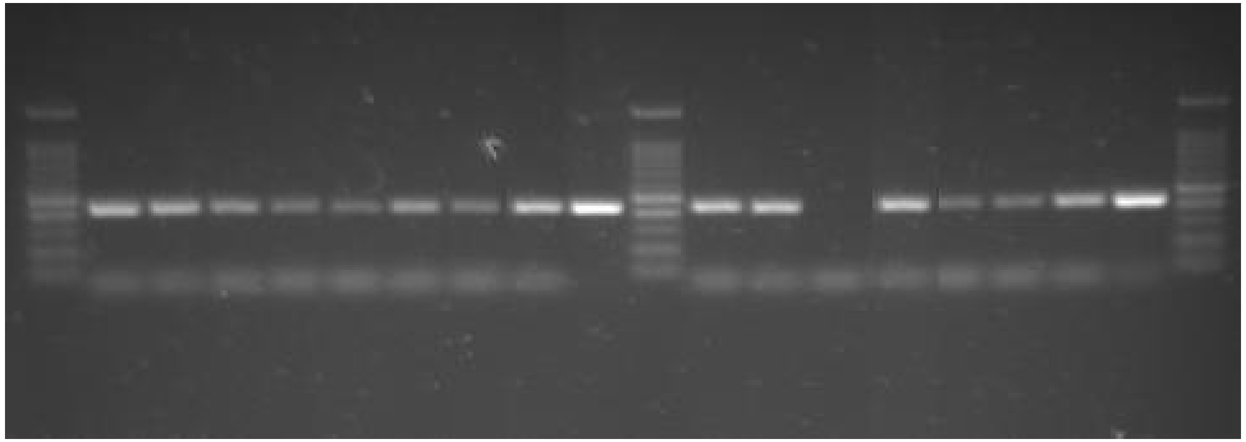

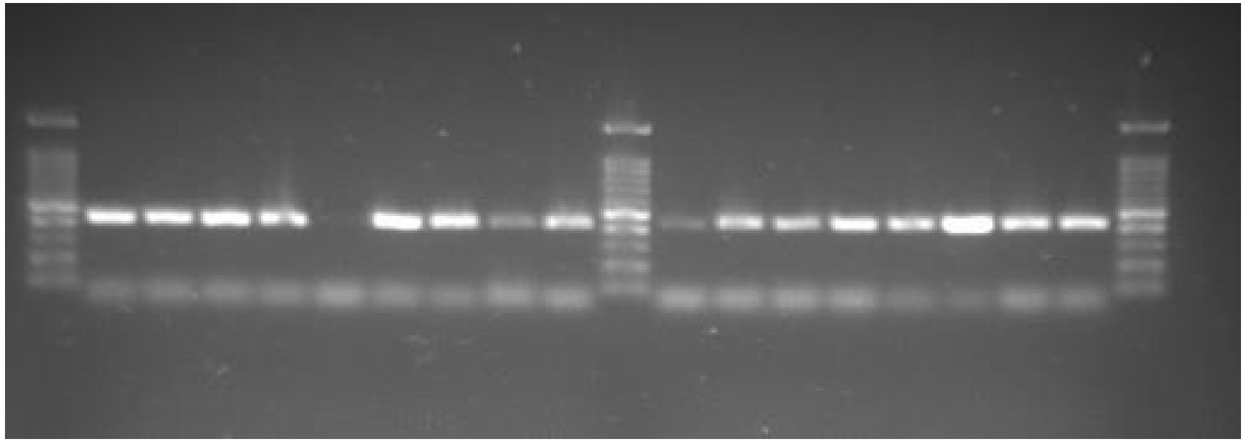

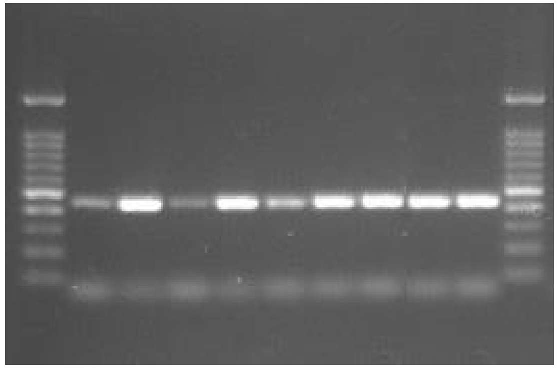

Number	Strain	Number	Strain
1	CBS7001	23	CBS431
2	CECT1369	24	NPCC1290
3	CECT10174	25	NPCC1309
4	CETC1969	26	NPCC1314
5	NCAIM676	27	NPCC1288
6	NCAIM789	28	NPCC1289
7	NCAIM868	29	NPCC1293
8	ZIM2113	30	NPCC1298
9	ZIM2122	31	NPCC1311
10	CECT12669	32	NPCC1317
11	CECT1189	33	NPCC1320
12	T73 (S.c.)	34	NPCC1321
13	S10	35	NPCC1322
14	S04	36	NPCC1323
15	S14	37	NPCC1324
16	S20	38	NPCC1328
17	CBS2898	39	NPCC1329
18	CBS2986	40	NPCC1330
19	CBS2946	41	NPCC1315
20	CBS377	42	NPCC1420
21	CECT10618	43	yHCT78
22	CECT12629		

Primers used: AB
(Chromosome VII)
