# Supplementary material for: Convergent adaptation of Saccharomyces uvarum to sulfite, an antimicrobial preservative widely used in human-driven fermentations
Source: PLoS Genet. 2021 Nov 11;17(11):e1009872. doi: 10.1371/journal.pgen.1009872 (PMC8631656; doi:10.1371/journal.pgen.1009872)
Supplement: S10 Fig — Two MBS concentrations (0.1 and 0.2 g/l) were evaluated. (PDF) [file pgen.1009872.s015.pdf]

0,1 g/l MBS

0,2 g/l MBS

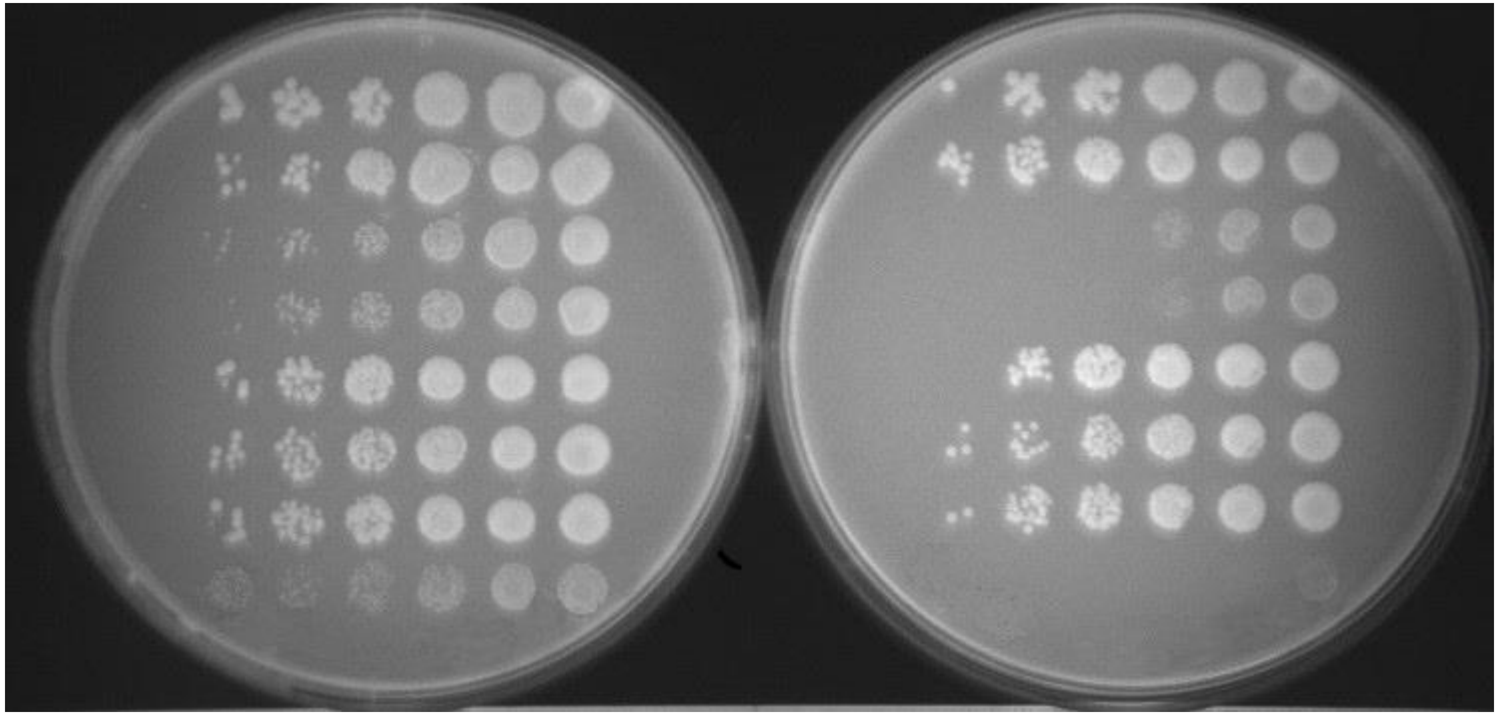

T73
BMV58
CBS7001(prBMV58) (replicate1)
CBS7001(prBMV58) (replicate2)
BR6-2
CBS7001(prBR6-2) (replicate1)
CBS7001(prBR6-2) (replicate2)
CBS7001
