# Supplementary material for: Convergent adaptation of Saccharomyces uvarum to sulfite, an antimicrobial preservative widely used in human-driven fermentations
Source: PLoS Genet. 2021 Nov 11;17(11):e1009872. doi: 10.1371/journal.pgen.1009872 (PMC8631656; doi:10.1371/journal.pgen.1009872)
Supplement: Attachment — Submitted filename: Response to reviewers.docx [file pgen.1009872.s017.docx]

The reviewers appreciated your findings and thought the conclusions were well supported. However, they also identified a number of issues that should be addressed. After reading the reviews I believe they have all given fair and thoughtful comments that can be addressed and will improve your work. I also have a few specific editorial comments.

1) One of the reviewers would like to see the work better placed into the larger picture of convergent evolution. I agree and believe it would be worth providing some context on how often convergence occurs through translocations and on a short time-scale. Convergence (or rather parallel evolution) in experimental evolution doesn't seem that relevant. But you may be able to argue your case is interesting because it involves translocations (which are quite rare) and occurred during a very short time interval since sulfites have been used.

The introduction has been remodeled to clarify the concept of convergent evolution and new examples have been included that describe this phenomenon in different organisms.

2) All three reviewers found the section on the selective sweep unclear in regards to the idea and how it is supported. After rereading this section I believe the intent is to show that the region around SSU1 VIItXVI has very little variation among the three strains having the translation, showing that the translocation is very young. A genome or chromosome plot of pairwise divergence would better convey this point. As it stands, the description of what was done/found is vague, e.g. "having more genes with nucleotide distances equal to zero". This could be 2 vs 1 gene which is not convincing. Also, why focus on coding sequences, which are often conserved, rather than all sequences extending from the breakpoint.

Unfortunately, this analysis can not be done at the whole genome or chromosome levels because our Illumina contig sequences do not cover whole chromosome and the gaps between contigs are different in the strains. Moreover, we tried to align the non-coding regions in the three strains and the alignment is possible in the conserved region between the two strains with the translocation but very difficult and with low significance when the other two strains, without translocation, are compared. Therefore, we decided to maintain the figure and better explain the purpose of the analysis of nucleotide divergence between strains, that is, to demonstrate that the translocation was originated in Europe and recently introduced in South America. The conservation of this region, including the translocation point, can be explained by a reduction of the recombination rate in the surrounding regions of the *SSU1* gene due to the translocation (as suggested by reviewer 1), but also by genetic hitchhiking (and not a selective sweep, as we incorrectly mentioned in the previous version of the manuscript) due to selection acting on the *SSU1* target. Both hypotheses are considered now in the discussion.

3) The reviewers also noted multiple areas where clarity and improvement in presentation could be made. And, they noted a number of points where there is missing information needed to replicating the study. In revising your work, please ensure that these relevant details are included. Also, please check the English for errors.

A new much more complete version of the manuscript is now presented that includes the suggestions of the reviewers, including new experiments, data and all the figures they asked.

Reviewer's Responses to Questions

**Comments to the Authors:
Please note here if the review is uploaded as an attachment.**

Reviewer #1: Macias et al, present a manuscript that describes the evolution of sulfite resistance by convergent mechanisms in S. uvarum. This evolution occurs by chromosome translocation that modifies the SSU1 promoter and promotes increased transcription level of the gene. Overall, there is not much to say about this paper as the results are fairly straightforward. Whether the results were unexpected is a matter of opinion - similar more extreme cases have been documented by Ken Wolfe’s lab on mating-type switching or by Sandy Johnson’s lab on mating-type specific expression though the exact mechanism of convergence may be different. Other simple aneuploidies have been observed throughout the tree of life as measures of adaptation.
Though most results support the conclusion of the paper, I find the presentation to be confusing. There are many sections in the result sections that don’t appear to have anything to do with the paper, and many sentences are stated without a clear interpretation of what the analysis means. I list here suggestions of locations where the text can be severely improved.
1) The section on phylogenetic reconstruction includes a rather large description of the phylogenetic tree. Every strain on the tree appears in the text, with a mention of their isolation source, their country of origin, and some hyperboles on the positioning of these strains. What is the meaning of all of this? Why can’t the authors just put the country/fermentation in the figure? It doesn’t seem like the large descriptive text from lines 220 to 240 to be useful.

As suggested, more information of the strains is now included in the figure 4 and the description of the figure has been shortened to highlight the most important aspects.

2) Line 257. I don’t understand what was done here. What is the SSU1 promoter of surrounding genes?

3) Line 262. A word appears to be missing here.

4) At the end of line 267, a concluding sentence is necessary here.

The whole paragraph was remodeled to better explain the analysis performed. We tried to better explain the purpose of the analysis of nucleotide divergence between strains, that is, demonstrate that the translocation was originated in Europe and recently introduced in South America.

5) I’m not familiar with how selective sweeps due to chromosomal rearrangements can occur. The sweeps the authors are talking about are due to sexual recombination, and I understand fairly well how a beneficial mutation can result in a sweep and loss of genetic diversity around the mutation. However, chromosomal rearrangements can often result in strange issues, such as mating isolation and non-viability. What is the proposed mechanism here for the spread of a translocation through sexual reproduction? Are there no essential genes in these translocated regions?
Is it possible that recombination is blocked at regions of chromosomal translocations, which would negate the evidence of a sweep in the traditional sense? Regions of inversions frequently block recombination, and it wouldn’t be surprising to me if it also occurred close to translocations.

Although recombination is blocked in regions with inversions (supergenes), the meiotic segregation of structurally normal chromosomes with translocated ones in heterozygous generates inviable meiotic products with duplicated and deficient regions, but recombination seems to be possible between chromosomes in a reciprocal translocation heterozygote. None the less, a reduction of the recombination rate in the surrounding regions of the translocated point would explain the conservation of *SSU1* segment between the translocated strains. Also a genetic hitchhiking (and not a selective sweep, as we incorrectly mentioned in the previous version of the manuscript) could also explain this conservation. Both hypotheses are now considered in the discussion.

6) What is GPY medium?

GPY is the general yeast media that was used in this study. The media definition was included in 4.1 section.

7) Figure 4: the 3 shades of grey for the types of chromosomal rearrangements should be changed. There’s no gradient needed for this (there is no difference in resistance for the two anyway).

As suggested, the symbols for the different rearrangements are now changed in the Figure 4.

8) Is it strange that SSU1 is not induced in the presence of MBS in WT strains but only induced in the strains with a chromosomal translocation? The authors have a large section on Fzf1 being the inducer of SSU1 but there is no evidence that this gene is even expressed in the WT strain (as shown in Fig 2 and 3).

As we have commented in lines 182-183, expression was significantly higher with MBS for all the strains, especially during the first two days of fermentation. Maybe the figure is confusing since the expression levels of the WT strains are much lower that the ones with the translocation. We have improved the Figure 2 to clarify that.

9) Figure 3 can be made clearer with a diagram of the promoter conformations that is being reconstructed.

We have added the diagrams to make the Figure 3 more clear.

10) There is a typo on figure 4 (“without”)

Corrected

11) Can the authors standardize their concentration units? Both molarity and mg/L are used for MBS. What is the typical concentration of sulfite in wine? Are the concentrations/resistance here relevant? How does the resistance compare to S. cerevisiae used in wine cultures?

We have standardized concentrations using g/l or mg/l. Concentration of sulfite in wine are typically 0.1-0.2 g/l, within the range of the concentrations used to test sulfite resistance in our work (0.1-0.4 g/l). A comment has been included in lines 158-159. The resistance is similar to *S. cerevisiae* strains with the translocation, a comment has been included in lines 162-164.

12) Line 192 to 210 is a word by word reconstruction of the graph we see in Figure 3. Can this be compressed somehow?

The paragraph was rewritten to compress and clarify the results.

Overall, some clarifications to the manuscript are required. Possible discussions of the caveats on the interpretation of results (#5), and further manuscript compression would make the paper clearer. Though I don’t disagree that parallel evolution to SSU1 overexpression has happened in S. uvarum, I think it is a bit of an exaggeration to say that the results were unexpected and I fail to appreciate how the findings here are placed in context with other modes of convergent evolution that have been previously observed in these fungal lineages.

Our conclusions were edited to avoid exaggerations and the manuscript has been modified to better transmit the statements taking into account the reviewers comments.

Reviewer #2: Macias et al. demonstrate an example of convergent evolution in adaptation to wine fermentation. Previous work has shown that wine strains of Saccharomyces cerevisiae have reciprocal translocation events that result in a chimeric promoter of the gene SSU1 and increase sulfite resistance. Here, the authors demonstrate a similar phenomenon in the distantly related species Saccharomyces uvarum, which is primarily used in wine and cider fermentation at low temperatures. They find two different translocation events in wine and cider strains of S. uvarum that also result in a recombinant SSU1 promoter, present in 11 different strains of this species. They demonstrate that strains with translocations have an increased tolerance to sulfite, and show that strains with the translocation have increased constitutive and inducible gene expression of SSU1. I think this manuscript highlights an interesting result of convergent evolution involving several independent translocations in S. cerevisiae and S. uvarum, but several conclusions could benefit from additional experiments, analysis, and explanation, elaborated below.

Major Comments
I had several questions related to the expression data. First, can the authors address the time sensitivity of the expression of SSU1? Why might it be elevated or decreased at different time points?

Besides Fz1p activation, *SSU1* regulation is mostly unknown although significant upregulation has been observed upon diauxic shift (Brauer MJ et al., 2005). Thus, we think that physiological changes that occur during the wine fermentation could alter *SSU1* expression.

What might be responsible for the drastic difference in expression of BMV58 between Figure 2 and 3?

The difference comes from a certain degree of variability observed in the *SSU1* expression data combined with the different normalization of the data. In figure 2, expression data is normalized with the data from strain NPCC1314 whereas in the figure 3, data is referred to CBS7001 strain as is detailed in the figure legends. This produces a clear visual difference in the BM58 expression but, in fact, unnormalized data showed no statistical differences between both datasets except time point 24 h in the experiment without MBS.

Second, the promoter expression experiments have a lot of variability between the promoter construct strain and the strain that it’s meant to mimic, particularly for the BMV58 strain. I think this data does support the conclusion that the translocation upstream of SSU1 influences expression, but the authors should further address potential discrepancies driving these differences (e.g., strain background differences, potentially not including all important regions in the promoter construct, etc.). Perhaps illustrating with a figure like Figure 1C what the promoter construct includes.

We agree with the reviewer that other unknown factors that are not included in the constructed strains CBS7001(prBR6-2) and specially CBS7001(prBMV58) might be influencing the upregulation of *SSU1* as is suggested from Figure 3. This is pointed out in the text at the end of the 2.3 section. To make more clear comparisons, schematic diagrams of the different *SSU1* locus are now included in the figure 3.

I would suggest to further clarify this, the authors could test sulfite tolerance in the CBS7001 wild type and the 2 promoter constructs.

As suggested we have compared test sulfite tolerance in the CBS7001 wild type and the 2 promoter constructs and a new figure S4 is now included. The results follow a similar trend than the *SSU1* gene expression results. A comment has been included at the end of the 2.3 section.

I’m having a hard time understanding the hypothesis of the selective sweep in relation to a translocation. The authors mention that the SSU1 translocation may be responsible for reproductive isolation between strains without the translocation, however, the strains with the translocation are not monophyletic. In Figure S2, the panel is showing a region that spans the translocation, but only one of the strains shown has the translocation, so I don’t understand what is being compared (e.g., for the strains without the translocation, is this comparing the syntenic regions on VII and XVI separately?). Inclusion of more strains in this analysis is needed (minimally all the other strains that have a translocation), and an extension beyond the described region, and including the other breakpoint for the translocation. More support and/or explanation is needed to strengthen this conclusion.

We tried to better explain the purpose of the analysis of nucleotide divergence between strains, that is, demonstrate that the translocation was originated in Europe and recently introduced in South America. The conservation of this region, including the translocation point, can be explained by a reduction of the recombination rate in the surrounding regions of the *SSU1* gene due to the translocation (as suggested by reviewer 1), but also by genetic hitchhiking (and not a selective sweep, as we incorrectly mentioned in the previous version of the manuscript) due to selection acting on the *SSU1* target. Both hypotheses are now considered in the discussion.

Minor comments
Introduction to convergent evolution - the examples provided from Arabidopsis and Drosophila are not what I would typically understand as convergent evolution. Can the authors elaborate more on their definition and how these examples illustrate the concept?

The introduction has been remodeled to clarify the concept of convergent evolution and new examples have been included that describe this phenomenon in different organisms.

Throughout the manuscript – I feel like the word “translocation” would be a better description of the events than “recombinations” (or alternatively chimeric promoter or recombinant promoter)

Corrected

Line 77- sensibility should be “sensitivity”

Corrected

Lines 155-157: Authors state that sulfite tolerance is significantly different between strains with the translocation and strains without, but no statistics are reported.

The statistical test and the significance are now included in the text and the figure.

Furthermore, the authors report that strains with the translocation can grow at 8mM sulfite concentration, but Figure 1D only shows up to 4 mM.

To standardize units, we are using g/l for MBS concentration across the manuscript and figures. The value of 8 mM was a mistake since it referred to sulfite but, in the experiments, we are using metabisulfite (4 mM), values are now corrected.

Perhaps a different figure type would be more appropriate for representation of this data, particularly because from this figure it appears that BR62- has the strongest tolerance to sulfite, but from my understanding, some of the strains with the other rearrangement have a higher tolerance.

Yes, other strains have higher tolerance. We think that this can be correctly interpreted with this figure since the error bar from the VII^XVI^ strains is higher than the bar for the BR6-2 strain.

Lines 175-176 – “carrying out the recombination VIIXVI” should be “with the translocation VIIXVI”

Corrected

193 – Delete, “Second,”

Corrected

206-210 – should clarify what they mean when they say unless the new promoter produces significant overexpression. Why would the chromosomal context or other elements not be important in the case of overexpression? And by overexpression, are they referring to specific time points like 96 hours?

We have rewritten the sentence to clarify the meaning.

*“*Although is clear that the new promoter of CBS7001(prBMV58) strain produces a significant overexpression of *SSU1* compared to the CBS7001 strain, these latter results suggest that, other factors as the chromosomal context or other unknown upstream/downstream elements, not transferred to CBS7001(prBMV58) could have further influenced *SSU1* expression in the BMV58 strain*”*

270 – “the case of a convergent adaptation of S. uvarum strains” to “a case of convergent adaptation of S. uvarum strains”

Corrected

287 – nucleotide divergence is ~20%

Corrected

289-291 – I’m not sure what the authors are trying to say with this sentence

Sentence was reformulated to clarify the meaning.

313 – “depending” should be “dependent”

Corrected

350-353 – rephrase to “Future studies should examine chromosomal rearrangements involving the gene responsible for sulfite detoxification in these species.”
Figure 4 – typo “Environments withouth sulfite”

Corrected

Reviewer #3: Macias et al report an interesting case of convergent adaptation to sulfite in various strains of S. uvarum. Resistance to sulfites induced by structural chromosomal changes leading to overexpression of SSU1 has been extensively studied in S. cerevisiae, but this manuscript represents the first description of this phenomenon in another species of Saccharomyces.

One major concern is that most of the raw results are not made available which makes difficult to assess the robustness of the analyses:
- The authors should provide the PCR amplification gels on the 61 strains as a supplementary figure.

The data is now included in the Figures S3A-F and summarized in table (Table S4). Thank to the suggestion of the reviewer we have identified two wrong identifications after the repetition of the PCR tests: strain CECT11036 is *S. cerevisiae* so it was not included in the final study and strain CECT12629 contains the VII^XVI^ translocation.

- The authors should also check by southern blot whether the rearrangement between chromosome XI and XVI corresponds to a reciprocal translocation event.

To check the rearrangement between chromosome XI and XVI we decided to perform PACBIO sequencing for the strain BR6-2 which is a technique that allows to perfectly identify chromosome rearrangements due to the long reads generated. The results effectively confirmed that the recombination exists and that it is reciprocal. A comment has been included at the end of section 2.1

- Similarly, the drop test assays on MBS should be presented.

The data is now included in the new Figure S5 and summarized in table S2.

- The CT values of the qPCR experiments should be made available.

A new supplementary table (Table S5) with the qPCR dataset is now included.

- How the substitutions of the WT SSU1 promoter by the BMV58 and BR6-2 SSU1 promoters were achieved in the reference background should be described with more details and controls.

We have expanded the description of the edited strain construction by CRISPR-Cas9 technique in section 4.2. The method was previously optimized for other studies and is routinely used in our lab.

In addition, it would be worth mentioning in the introduction that nearly 600 S. cerevisiae strains from fermented grape juice were genotyped for the presence of the reciprocal translocation between chromosomes VIII and XVI (Marullo et al. Front Microbiol, 2020). Another interesting finding that could be worth mentioning is that in the absence of a pre-existing triplication in the ECM34 promoter, this translocation was shown to promote sulfite sensitivity in the BY laboratory strain background (Fleiss et al. Plos Genet 2018).

The suggestions were included in the introduction.

There are other questions, listed below, that would deserve to be answered before this manuscript is suitable for publication.
- In SupTable 1, the number of annotated genes ranges from 1369 to 5666. Where does such a large discrepancy come from?

The difference comes from the quality and the coverage of the sequencing. Some of the assemblies that have the lowest number of genes annotated are coming from the paper Almeida et al. (2014), where these particular strains (e.g. CRUB1778, CRUB1779 and CRUB1586) had very low sequencing coverage. We annotated the assemblies and extracted only the gene sequences from those assemblies that correspond to genes without gaps. Some of the assemblies were very fragmented and this is the reason why we get lower number of genes to perform the phylogeny.

- In addition, the sequencing and assembly statistics for the 21 genomes should be provided (coverage, N50, number of contigs, nb of scaffolds, % of the reference genome covered, etc).

We have included extra columns in table S1 showing the assembly statistics.

- One general question about the 21 sequenced strains would be to know their ploidy and their heterozygosity levels because diploid genomes could be heterozygous for the translocations. Given that a single allele can only be represented in the genome assemblies, it is possible that other cases of translocation remain undetected.
- Line 115: The authors should better explain how they identified the rearrangements “Assemblies allowed us to identify two candidate chromosomal rearrangements in the promoter of this gene located at chromosome XVI”

We assembled and annotated our strains using a combination of two approaches (one of them is based on synteny conservation). We perform a quick step of manual correction once the annotations are made. During our manual curation we observed the *SSU1* gene annotated at a different chromosome than XVI. A new comment is now included to detail this in the manuscript.

- Line 129 : change VIItXVI by VIIItXVI

Corrected

- In Fig 1C, the interesting information would not so much be the identity of the gene upstream SSU1 after the translocation but rather the identity of the new promoter brought in front of SSU1 by the rearrangement. Are they any genome-wide expression data available that would allow to look for the expression pattern of these promoters in a WT configuration?

We have performed prelaminar gene expression studies with a few strains and time points during winemaking that include the strain BMV58, that contains the VII^XVI^ translocation and other *S. uvarum* strains without the translocation and we observed an overexpression of *SSU1* gene. The expression pattern is similar to what we have observed in Figure 2.

- Line 256: The authors propose that a selective sweep of 117 kb occurred surrounding the SSU1 promoter region between the NPCC1417 and BMV58 strains. I don’t think that the term selective sweep is well chosen because it is hard to imagine that selection was able to eliminate all the genetic diversity across such a large chromosomal segment. The conservation between the chromosome configuration and the DNA sequence between the 2 strains most likely result from a recent admixture between the 2 backgrounds as suggested in the discussion. In addition, the author should also provide a hypothesis on the evolutionary origin of the translocation in the CECT12600 strain.

We tried to demonstrate that the translocation was originated in Europe and recently introduced in South America by recent admixture, in which genome segments were shuffled due to recombination. However, the conservation of this large block including the translocation point, can not be simply explained by admixture and recombination according to the distribution of pairwise nucleotide diversity in randomly selected windows of the same size (p-value < 0.05; Whitney-Wilcoxon test). Therefore, the conservation of such a large segment could be explained by a reduction of the recombination rate in the surrounding regions of the *SSU1* gene due to the translocation (as suggested by reviewer 1), but also by genetic hitchhiking (and not a selective sweep, as we incorrectly mentioned in the previous version of the manuscript) due to selection acting on the *SSU1* target. Both hypotheses were considered in the discussion.

Finally, the word ‘recombination’ is not correctly used throughout the text and should be replaced by ‘rearrangement’ in many instances.

Corrected

In general, I think the English could be improved.

A detailed revision of the whole manuscript was performed to correct and improve language use and style.
