# Supplementary material for: Convergent adaptation of Saccharomyces uvarum to sulfite, an antimicrobial preservative widely used in human-driven fermentations
Source: PLoS Genet. 2021 Nov 11;17(11):e1009872. doi: 10.1371/journal.pgen.1009872 (PMC8631656; doi:10.1371/journal.pgen.1009872)
Supplement: Attachment — Submitted filename: Response to reviewer.docx [file pgen.1009872.s018.docx]

Reviewer #1: The revised manuscript has addressed all my previous concerns.
I have a few comments for clarity:
1) Figure 3: The orders of the bars on the histogram should be the same order as the legend below.

Corrected

2) Figure S2 is missing from this submission

Corrected

Reviewer #2: I appreciate that the authors added several new components to their manuscript to address reviewer concerns (including PacBio sequencing to identify reciprocal translocation, sulfite tolerance of CBS7001 and promoter constructs, etc), and I like the new addition to Figure 3.

The idea that recombination is reduced around the translocation breakpoints is logical to me, and I don’t want to belabor this, but I think the analysis presented and the response to reviewers on this topic could still use further work. At the least, the same analysis should be conducted at the other breakpoint of the translocation for the given strains. If my understanding is correct, we would predict that the other breakpoint would show a similar signature.

As suggested we have performed the analysis on the other translocated chromosome and the results showed a clearly shorter region of low genetic divergence around the breakpoint, which supports the action of selection on the new *SSU1* allele. A new figure (Figure S3) is now included, and a comment is now introduced in section 2.4.

This section could use more support from the literature about what is known/expected about patterns of recombination and diversity around breakpoints. Ideally other strains with the same translocation would be compared to identify if the same blocks are conserved. I do not see how the presented data could be consistent with hitchhiking, so if the authors think this is plausible, they need to elaborate on this point.

Genetic hitchhiking is the increase in frequency of an allele attributable to positive natural selection acting on another site linked to it. By using the coalescent process for a random sample of genes from a selective neutral locus linked to a locus at which selection is acting, a stochastic, finite population model was developed by Kaplan, Hudson and Langley (1989, Genetics 123: 887-899). This model described the steady-state effect of hitchhiking on the distribution of the number of selectively neutral polymorphic sites in a random sample. The model predicts that, in regions of low crossing-over, strongly selected substitutions in the history of the sample can substantially reduce the number of polymorphic sites in a random sample of genes from that expected under a neutral model, because linked regions of the genome are swept along with the selectively favored mutant as it increases in frequency and eventually fixes in the population. The establishment of the boundaries of the region affected would make it possible, based on their mathematical model, to calculate both the age and the strength of selection associated with the selective sweep (as an example see Sáez et al. 2003, PNAS 100:1793–1798). In the present study, the low number of sequences did not allow to perform a similar approach, but an outcome of the hitchhiking model is that, under the same crossing-over rate and age, the longest the region affected, the largest the strength of selection.

The fact that the conserved region surrounding the reciprocal translocation site is significantly smaller, within the range expected by random, does not support a lower recombination rate in the regions flanking the translocation point and, hence, is compatible with the presence of a large linked region swept along with the selectively favored recombinant *SSU1* allele.

This last paragraph has been introduced in the discussion section.

A few other minor points:
I have a better understanding of the presented expression data now. I appreciate the authors answering my queries regarding the time course and variation in expression, as well as the differences between Fig. 2 and 3. I would suggest that the authors incorporate what is known about the time course and expression into the text.

A new comment is included at the end of the section 2.3 to explain the differences observed between figures 2 and 3 with respect to the BMV58 strain.

I have one additional comment here – the figure legends read 15 mg/L concentration but most other assays are done with a concentration 10x that or more, including the newly added Figure S4. From Figure S4, the promoter construct CBS7001(prBMV58) clearly cannot explain sulfite resistance in higher levels of BMS. I see the authors note this in the results, might also be worth mentioning more clearly that expression was not tested at high concentrations.

A new comment regarding on this is now included at the beginning of the 2.3 section.

A couple of the supplementary files mentioned in the text seem to be missing from this version (Figures S2, S5)

Corrected

I would suggest the authors include in the materials and methods or in their Table S1 the assembly statistics for the PacBio sequencing and if anything was done besides manual inspection to confirm the reciprocal translocation.

The statistical parameters of the PacBio sequencing and assembly are now included in the Table S1.

The paragraph on convergent evolution in the introduction is improved, but could still use a bit more tweaking. I have included it here edited for grammar, and suggest that the authors try to clarify more in their examples what they mean by convergent evolution. For example, in the insect example to toxic compounds, the authors could state what molecular mechanisms were found to underlie this adaptation, and thus how it was concluded that different lineages independently evolved this phenotype. In the yeast examples, the authors could specify with more details, such as point mutations in different genes resulted in the same phenotype - increased tolerance to X, etc.

Organisms belonging to different lineages can evolve independently to overcome similar
environmental pressures through different molecular mechanisms. This phenomenon, known as convergent evolution, is considered evidence of the action of natural selection [1,2]. In recent
years, comparative genomics studies have suggested that convergent adaptation occurs
more frequently than previously expected [3,4]. For example, species of insects spanning multiple
orders have independently evolved higher tolerance to toxic compounds
produced by plants [5], demonstrating that convergent adaptation can
occur in nature between organisms belonging to different taxonomic levels. In the case
of yeasts, convergent evolution by point mutations has been described both in evolving
yeast species in nature [6] and in short-term evolutionary studies in the species
Saccharomyces cerevisiae [7], for example in populations evolved under glucose
limitation [8]. Convergent evolution can occur through different mechanisms, including point
mutations, gene duplications, and interspecific hybridization. Examples of convergent evolution via chromosomal rearrangements are rare, a single study has suggested that an intrachromosomal translocation is responsible of a convergent evolution in independent lineages in the case of the major histocompatibility complex [9]. A second study has suggested that amylase evolution in fish may have converged though a putative chromosomal translocation, although this has not yet been confirmed [10].

We appreciate the effort of the reviewer to correct himself the paragraph. We have included all his/her suggestions to clarify and optimize this part of the introduction.

Reviewer #3: The authors have responded satisfactorily to all my requests. I am wondering though whether the new PacBio data were made publicly available.

The PacBio data is already included in the BioProject PRJNA471597 that will be available after the publication of the study.
